# PATH ANALYSIS OF EDUCATION AND DISEASE BURDEN IN DEMENTIA VULNERABILITY

**DOI:** 10.1093/geroni/igz038.2589

**Published:** 2019-11-08

**Authors:** Sarah B Hubner, Hyeon Jung Kim, Julie Blaskewicz Boron

**Affiliations:** University of Nebraska at Omaha, Omaha, Nebraska, United States

## Abstract

When considering the various extrinsic variables that may affect disease vulnerability, it is valuable to study temporal ordering of factors to identify areas for disease intervention efforts. This study sought to inform improvement of networks for the purposes of education and health by attempting to better define the causal ordering of ethnicity, age, gender, education, disease burden, and dementia diagnosis with the Aging, Demographics, and Memory Study, a sub-study of the Health and Retirement Study. Participants and/or proxies self-reported total number of chronic conditions, subsequently regarded as disease burden. Assessments occurred over four waves; participants were not reassessed after dementia diagnosis. The current study categorized participants as demented (n=414), identified in any wave, or normal (n=117), identified in the final wave. Cognitively-impaired-not-demented and deceased participants were not considered due to lack of diagnosis. Cross-sectional weighting was used. A path model was developed; ethnicity, age, and gender were antecedent to education, and education was casually ordered before disease burden, which was antecedent to dementia diagnosis. A series of logistic and linear regression analyses were conducted. Results revealed that being non-white (Σβ=0.643, all p’s<.001), of older age (Σβ=0.250, all p’s<.001), female (Σβ=0.180, all p’s<.001), and having increased disease burden (Σβ=0.118, p<.001) all demonstrated a positive total effect on dementia diagnosis. Conversely, more years of education (Σβ=-0.245, p<.001) had a negative total effect on diagnosis. The education to disease burden pathway was non-significant. Ultimately, these results may indicate a need for dementia interventions that target those with low education or high disease burden.

